# A Reverse Genetics System for Zika Virus Based on a Simple Molecular Cloning Strategy

**DOI:** 10.3390/v10070368

**Published:** 2018-07-12

**Authors:** Maximilian Münster, Anna Płaszczyca, Mirko Cortese, Christopher John Neufeldt, Sarah Goellner, Gang Long, Ralf Bartenschlager

**Affiliations:** 1 Department of Infectious Diseases, Molecular Virology, Heidelberg University, Centre for Integrative Infectious Disease Research, Im Neuenheimer Feld 344, 69120 Heidelberg, Germany; max.muenster1991@googlemail.com (M.M.); anna.plaszczyca@med.uni-heidelberg.de (A.P.); mirko.cortese@med.uni-heidelberg.de (M.C.); Christopher.Neufeldt@med.uni-heidelberg.de (C.J.N.); sarah.goellner@med.uni-heidelberg.de (S.G.); 2 Key Laboratory of Molecular Virology and Immunology, Institute Pasteur of Shanghai, Chinese Academy of Sciences, Shanghai 200031, China; glong@ips.ac.cn; 3 German Center for Infection Research, Heidelberg Partner Site, Im Neuenheimer Feld 344, 69120 Heidelberg, Germany

**Keywords:** ZIKV, reporter virus, cryptic promoter silencing, full-length molecular clone, subgenomic replicon, plasmid toxicity

## Abstract

The Zika virus (ZIKV) has recently attracted major research interest as infection was unexpectedly associated with neurological manifestations in developing foetuses and with Guillain-Barré syndrome in infected adults. Understanding the underlying molecular mechanisms requires reverse genetic systems, which allow manipulation of infectious cDNA clones at will. In the case of flaviviruses, to which ZIKV belongs, several reports have indicated that the construction of full-length cDNA clones is difficult due to toxicity during plasmid amplification in *Escherichia coli*. Toxicity of flaviviral cDNAs has been linked to the activity of cryptic prokaryotic promoters within the region encoding the structural proteins leading to spurious transcription and expression of toxic viral proteins. Here, we employ an approach based on in silico prediction and mutational silencing of putative promoters to generate full-length cDNA clones of the historical MR766 strain and the contemporary French Polynesian strain H/PF/2013 of ZIKV. While for both strains construction of full-length cDNA clones has failed in the past, we show that our approach generates cDNA clones that are stable on single bacterial plasmids and give rise to infectious viruses with properties similar to those generated by other more complex assembly strategies. Further, we generate luciferase and fluorescent reporter viruses as well as sub-genomic replicons that are fully functional and suitable for various research and drug screening applications. Taken together, this study confirms that in silico prediction and silencing of cryptic prokaryotic promoters is an efficient strategy to generate full-length cDNA clones of flaviviruses and reports novel tools that will facilitate research on ZIKV biology and development of antiviral strategies.

## 1. Introduction

The Zika virus (ZIKV), a member of the *Flavivirus* genus within the *Flaviviridae* family, was identified almost 70 years ago in Uganda [1] but until recently was not associated with severe symptoms. However, outbreaks outside of Africa and Asia, in the Yap Islands (2007) [2], French Polynesia (2013) [3] and the Americas (2015) [4], raised major interest as infection was associated with an increased incidence of microcephaly and other neurological manifestations in developing foetuses as well as Guillain-Barré syndrome in infected adults [5,6]. Two lineages of ZIKV have been identified, African and Asian, with the currently circulating strains belonging to the Asian lineage [7]. Although 48 countries have confirmed ZIKV infections associated with *Aedes* mosquito-borne transmission of the virus, neither a prophylactic vaccine nor antiviral therapies are available to date [8]. As a consequence, there is an urgent need for tools, which facilitate studying the molecular determinants that underlie ZIKV pathogenesis and allow testing of potential antiviral therapies. In this respect, stable and traceable reverse genetic systems to generate isogenic mutants, are of great advantage [9,10]. However, construction of ZIKV molecular clones has been hampered by the instability of the viral cDNA genome during propagation via bacterial plasmids. The instability of flaviviral cDNA clones in *Escherichia coli* (*E. coli*) has been linked to the expression of toxic viral proteins from cryptic *E. coli* promoters (CEPs) encoded in the flavivirus genome [11,12]. Strategies to disrupt toxic protein expression and to overcome these toxicity problems include insertion of introns into the viral open-reading frame [13,14] or propagation of ZIKV genome fragments on multiple plasmids and subsequent assembly of the fragments [15,16,17,18,19]. Those strategies, however, have some disadvantages compared to a single plasmid system that allows in vitro transcription of full-length infectious viral RNAs. For instance, the intron insertion method requires nuclear transcription to generate the viral genome with the risk of undesired splicing rendering the RNA non-functional. In the case of multi-plasmid systems, laborious and potentially error prone in vitro assembly steps complicate the protocol. Although Shan and co-workers were able to amplify a full-length ZIKV cDNA clone from Cambodia (FSS13025 strain, isolated in 2010) on a single plasmid in *E. coli*, ours and others’ data indicate that this is neither possible for the prototypic African strain MR766 nor for the French Polynesian strain H/PF/2013 (Asian lineage) or isolates from the Americas [13,15,16,17].

Here we present a different approach that overcomes these problems and is based on the observation that full-length Japanese encephalitis virus (JEV) and Dengue virus 2 (DENV2) infectious cDNAs could be stabilized by CEP silencing [11]. We show that mutational inactivation of multiple CEPs predicted in silico to reside in the structural regions of the MR766 and H/PF/2013 genomes is sufficient to stabilize the full-length cDNA genomes of both ZIKV strains enabling the construction of a single-plasmid based reverse genetic system. Authentic virus genomes and engineered reporter viruses generated with this approach are fully functional in cell culture and suitable for multiple research and development purposes.

## 2. Materials and Methods

### 2.1. Cell Lines and Antibodies

VeroE6 and Huh7 cells were cultured at 37 °C and 5% CO_2_ in Dulbecco’s modified Eagle’s medium (DMEM) (Life Technologies, Darmstadt, Germany), supplemented with 10% foetal calf serum (FBS; Sigma-Aldrich, Taufkirchen, Germany), 2 mM L-glutamine, nonessential amino acids (all from Gibco, Life Technologies, Darmstadt, Germany), 100 U/mL penicillin and 100 μg/mL streptomycin (DMEMcplt). Primary antibodies used in this study were: rabbit anti-ZIKV NS3 and anti-NS4B (both from GeneTex, Irvine, CA, USA), mouse pan-flavivirus-anti-E (4G2, ATCC^®^, Manassas, VA, USA), mouse J2 anti-dsRNA antibody (Scicons, Szirák, Hungary), mouse anti-glyceraldehyde-3-phosphate dehydrogenase (GAPDH, Santa Cruz Biotechnology, Heidelberg, Germany). Secondary horseradish peroxidase-conjugated antibodies were purchased from Sigma-Aldrich. AlexaFluor-conjugated secondary antibodies were obtained from Life Technologies.

### 2.2. Source of Virus Sequences

For the construction of the ZIKV genome we used the reference genomes KJ776791 (H/PF/2013, accession date August 2016) and DQ859059 (MR766, accession date August 2016). In addition, we amplified the MR766 and the H/PF/2013 strains (obtained from the European Virus Archive; Marseille, France) by passaging once in C6/36 mosquito cells and once in VeroE6 cells. Viral RNA was isolated from cell lysates using the NucleoSpin RNA II kit (Machery-Nagel, Düren, Germany) and reverse transcribed using SuperScript III RT (Thermo Fisher Scientific Waltham, MA, USA). cDNA was amplified by PCR and amplicons were sequenced by Sanger sequencing (GATC Biotech, Constance, Germany) using primers spanning the complete ZIKV genome. Sequences of the 5′ and 3′UTRs were obtained by the rapid amplification of cDNA ends (RACE) using the 5′/3′ RACE second generation kit (Roche, Basel, Switzerland) with a polyA-tail added to the cDNA prior to the 3′ RACE reaction by using the poly(A) polymerase (New England Biolabs, Ipswich, MA, USA).

### 2.3. In Silico Prediction of CEPs and Sequence Modifications

Cryptic *E. coli* promoters were predicted with the publicly available Neural Network promoter program from the Berkeley Drosophila Genome Project [20,21] similar to an earlier report [11]. The ZIKV sequences were analysed for CEPs from nucleotide position 1–2683 for H/PF/2013 and 1–2664 for MR766. Putative promoters with a score >0.85 were eliminated by silent nucleotide exchanges introduced into the −10 regions (Pribnow/Schaller box) and/or the −35 regions (Figure S1). CEPs in the 5′UTR were not modified to avoid changes in RNA secondary structures (Acosta et al., 2014 [22]). In addition, in order to facilitate assembly and reverse genetic studies, several restriction sites were inserted or removed by silent nucleotide exchanges (Tables S1 and S2). The T7 promoter sequence (5′-TAATACGACTCACTATAG-3′) was inserted upstream of the 5′UTR to allow for in vitro transcription of viral RNA. The final sequences were re-analysed with the Neural Network promoter program to confirm that the scores were below 0.85. The sequences were ordered as synthetic DNA fragments (four fragments/strain) from the GeneArt Gene Synthesis service (Invitrogen, Darmstadt, Germany).

### 2.4. Generation of synZIKV Constructs

**Figure 1:**
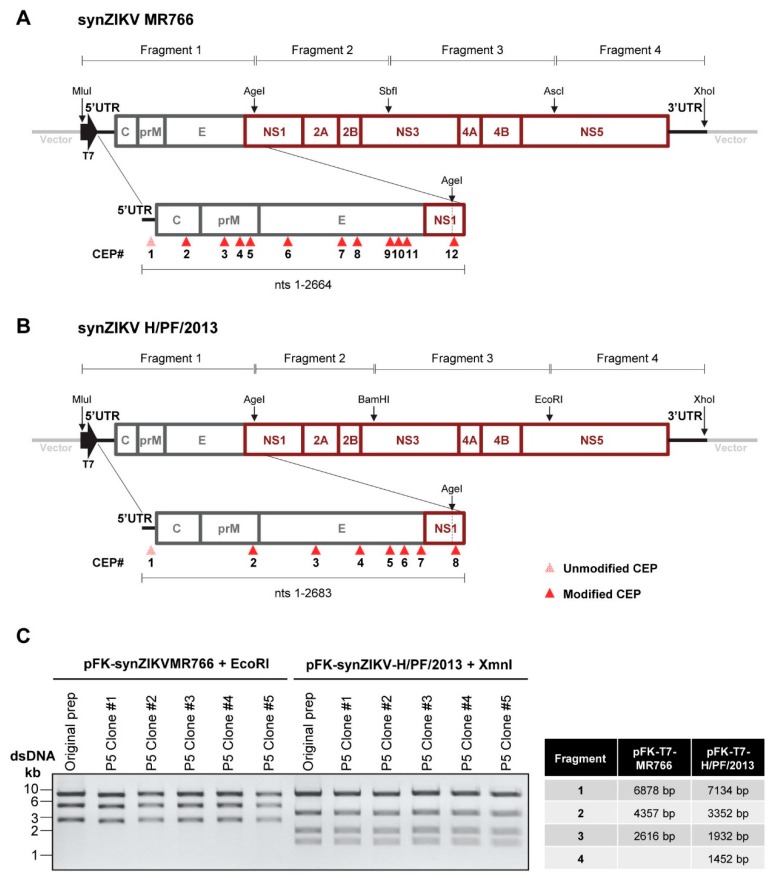
Construction and stability of synthetic full length Zika virus (synZIKV) cDNA clones. (**A**) Schematic representation of the synZIKV MR766 construct and the four fragments used to assemble the genome. The 5′ and 3′UTRs are indicated with bold black lines, the promoter for the T7 RNA polymerase with a black arrow. Restriction sites used for the assembly of the fragments are indicated. An enlargement of fragment #1 is shown below with putative CEPs (score > 0.85) indicated by red arrow heads. CEP 1 was not mutated (indicated with the pink arrow head). (**B**) Same as in panel (**A**) but for synZIKV-H/PF/2013. (**C**) Restriction patterns of pFK-synZIKV constructs obtained after digest with EcoRI (MR766) or XmnI (H/PF/2013) and agarose gel electrophoresis. Plasmids were analysed directly after assembly (original prep) and after five passages (P5) in *E. coli* (five DNA clones of P5 are shown).

The pFK vector used for the assembly of the synthetic ZIKV (synZIKV) sequences has been described previously (Lohmann et al., 1999 [23]). A synthetic DNA linker encoding the restriction sites required for assembly of the synZIKV cDNA clones (Figure 1A) was inserted into the vector via HindIII and SpeI. For assembly of the full-length wild-type synZIKV plasmids (pFK-synZIKV) the synthetic DNA fragments were inserted into the modified pFK vector using the indicated restriction enzymes (Figure 1A) and in four steps in the order of fragment 4 to fragment 1. Plasmids were amplified in dcm^+^/dam^+^ DH5α cells. To generate full-length *Renilla* luciferase (RLuc) reporter constructs (pFK-synZIKV-R2A), we used a construct design similar to the one reported by us for the synthetic DENV-2 16681 reporter genome [10]. In brief, we constructed a synthetic DNA fragment encoding the T7 promoter followed by the 5′UTR of ZIKV, the first 102 nts of Capsid required for genome circularization, the *RLuc* gene flanked by NotI and NruI restriction sites and the auto-proteolytic FMDV 2A peptide directly fused to the first nucleotide of the ZIKV coding region. This fragment was inserted into the pFK-synZIKV plasmid via MLuI/KpnI restriction sites. Sub-genomic synZIKV RLuc reporter replicons (pFK-synZIKV-sgR2A) were constructed in an analogous way but with the difference that the reporter cassette was inserted between the last 24 codons of E that we retained to ensure proper membrane insertion of NS1 (cloning via MLuI and AgeI restriction sites). To generate turbo far-red fluorescent protein FP635 expressing viruses (pFK-synZIKV-FP635 constructs), the reporter gene was amplified by PCR using the FP635-encoding DENV2 16681 construct reported earlier [24] and inserted into the pFK-synZIKV-R2A plasmids via the NotI and NruI restriction sites flanking the *RLuc* gene. Note that a coding sequence for the SV40 NLS (PKKKRKV) was fused in frame to the 3′end of the turbo far-red fluorescent protein FP635-encoding sequence by using PCR (primer sequences available on request). All nucleotide sequences of the final constructs were validated by using Sanger sequencing (GATC Biotech).

### 2.5. In Vitro Transcription and RNA Transfection

The protocol for in vitro transcription has been described earlier [23]. Briefly, synZIKV sequences were linearized with XhoI (located at the end of the 3′UTR of the viral genome) and the DNA purified with the Nucleo-Spin Extract II kit (Macherey-Nagel, Düren, Germany). The in vitro transcription reaction was carried out with 10 μg of linearized plasmid DNA in a total volume of 100 μL containing 20 μL 5× RRL buffer (400 mM HEPES (pH 7.5), 60 mM MgCl_2_, 10 mM spermidine and 200 mM DTT), NTP-Mix (3.125 mM ATP, CTP and UTP and 1.56 mM GTP), 1 U/μL RNasin (Promega, Madison, WI, USA), 2 U/μL T7 RNA polymerase (New England Biolabs) and 1 mM anti-reverse cap analogue (ARCA; 3′-O-Me- m7G(5′)ppp(5′)G; New England Biolabs). After incubation at 37 °C for 2.5 h, 1 U/μL T7 RNA polymerase was added followed by additional 2.5 h incubation at 37 °C. DNA was digested with DNaseI for one hour and RNA was purified by acidic phenol-chloroform extraction and isopropanol precipitation. The integrity and size of the RNAs was validated by agarose gel electrophoresis. For electroporation, subconfluent and trypsinized cells were collected in DMEMcplt, washed once with PBS and resuspended in cytomix buffer (120 mM KCl, 0.15 mM CaCl_2_, 10 mM potassium phosphate buffer, 25 mM HEPES (pH 7.6), 2 mM EGTA, 5 mM MgCl_2_, freshly supplemented with 2 mM ATP and 5 mM glutathione) at a concentration of 1 × 10^7^ cells/mL for Huh7 and 1.5 × 10^7^ cells/mL for VeroE6 cells. Four hundred μL of the cell suspension was mixed with 10 μg of in vitro transcribed RNA, transferred into an electroporation cuvette (Bio-Rad, Hercules, CA, USA; 0.4-cm gap width) and pulsed once with a Gene Pulser II system (Bio-Rad) at 975 μF and 270 V. Finally, the cells were transferred into pre-warmed DMEMcplt in case of synZIKV-sgR2A replicons or DMEMcplt supplemented with 15 mM HEPES (pH 7.5) in case of the full-length synZIKV. For replication assays, Huh7 cells transfected with synZIKV-sgR2A RNAs were seeded into 12-well plates at a density of 2 × 10^5^ cells/well. VeroE6 cells transfected with full-length synZIKV RNAs were seeded into 24-well plates at a density of 2 × 10^5^ cells/well.

### 2.6. Virus Stocks and Passaging

Stocks of parental ZIKV strains were produced exactly as described [25]. For production of wild-type synZIKV stocks, two electroporation reactions of the same construct were pooled in 20 mL DMEMcplt and seeded into a single 15 cm-diameter dish. After 48 h, the medium was changed to DMEMcplt containing 15 mM HEPES (pH 7.5). Supernatants were harvested at least twice (between 72–96 h for synZIKV-MR766 and 96–120 h for synZIKV-H/PF/2013). Virus-containing cell culture supernatants were filtered through a 0.45 μm syringe filter and plaque-forming units (PFU) were determined. For final stock production, 7 × 10^6^ VeroE6 cells were seeded into 15 cm-diameter dishes and infected at a multiplicity of infection (MOI) of 0.1 on the next day. Infected cells were cultured in DMEMcplt containing 15 mM HEPES (pH 7.5) and supernatants were collected from day 3–7 post-infection as described above. Aliquots of the virus stocks were stored at −80 °C. For cell culture adaptation of the synZIKVs, multiple rounds of infection were performed in Huh7 cells. Virus stocks (passage 0; P0) were prepared as described above and used to infect Huh7 cells at MOI = 0.1. Virus containing supernatants were harvested at 72 h post infection (P1) and passaged two more times in 72 h hour intervals (P2–P3).

### 2.7. Virus Titration by Plaque Assay

For titration of wild-type viruses, VeroE6 cells were seeded into 24-well plates at a density of 2.5 × 10^5^ cells/well one day prior to infection. The cells were infected with serial 10-fold dilutions of virus containing supernatants for one hour at 37 °C. All plaque assays were performed in duplicates. After infection, the inoculum was removed and replaced with serum-free MEM (Gibco, Life Technologies) containing 1.5% carboxy-methylcellulose (Sigma-Aldrich). After four days, cells were fixed by the addition of 5% formaldehyde for at least 2 h at room temperature. Fixed cells were washed with water and stained with 1% crystal violet in 10% ethanol for at least 15 min. After rinsing the cells with water, the number of plaques was counted and virus titres were calculated as plaque forming units/mL (PFU/mL).

### 2.8. RLuc Assays

RLuc activity was determined as previously described [26]. At the indicated time points cells were lysed by addition of 100 μL (full-length synZIKV-R2A) or 125 μL (synZIKV-sgR2A replicons) luciferase lysis buffer (25 mM Glycine-Glycine (pH 7.8), 15 mM MgSO_4_; 4 mM EGTA, 10% (*v*/*v*) glycerol, 0.1% (*v*/*v*) Triton X-100, freshly added 1 mM DTT) to each well. The lysates were stored at −80 °C until use for luciferase assays. Luciferase activity was determined with a Lumat LB9507 luminometer (Berthold Technologies, Bad Wildbad, Germany). For each sample, 20 μL of cell lysate were mixed with 100 μL freshly prepared luciferase assay buffer (25 mM Glycine-Glycine (pH 7.8), 15 mM K_4_PO_4_ buffer (pH 7.8), 15 mM MgSO_4_, 4 mM EGTA, 1.42 μM coelenterazine).

### 2.9. Antiviral Assays and Stability of synZIKV-R2A Viruses

For characterization of RLuc-encoding synZIKV-R2A clones, VeroE6 cells transfected with the respective in vitro transcripts were seeded into 24-well plates at densities of 2 × 10^5^ cells/well. Supernatants were collected 72 h post-electroporation (Passage 0; P0) and stored at −80 °C until use for antiviral assays or further passaging. For antiviral assays VeroE6 cells were seeded into 24-well plates at a density of 1 × 10^5^ cells/well and on the next day infected with a 1:10 dilution of P0 of the respective virus at 37 °C. One hour later the inoculum was removed and replaced with DMEMcplt containing the indicated concentrations of 2′-C-methylcytidine (2′CMC; Sigma-Aldrich). RLuc activities were determined 72 h post-infection. For assessment of the stability, synZIKV-R2A P0 reporter viruses were subjected to multiple rounds of infection of VeroE6 cells (72 h infection/passage). To determine reporter virus stability, Huh7 cells were seeded into 24-well plates at a density of 7.5 × 10^4^ cells/well and infected on the next day with supernatants from each passage as described above. After 72 h supernatants were collected and subjected to plaque assay analysis. Cells were lysed in luciferase lysis buffer and RLuc activities were determined as described above. To check for the integrity of the reporter genomes, RNA was isolated from P0–P3 virus-containing supernatants using NucleoSpin RNA II kit (Machery Nagel, Düren, Germany), reverse transcribed with SuperScript III RT using random hexamer primer (Thermo Fisher Scientific) and amplified by PCR using the forward primer 5′CGACAGTTCGAGTTTGAAGC3′ hybridizing to the 5′UTR of both strains and the reverse primers 5′AGGCTAGAATCGCCAAGACC3′ and 5′GTTGATGAGGCCCAGTGATG3′ complementary to the capsid coding region of H/PF/2013 and MR766, respectively. Amplicons were analysed by agarose gel electrophoresis using Midori Green (Biozym, Hessisch Oldendorf, Germany) staining of DNA.

### 2.10. Immunofluorescence Microscopy and Western Blotting

For immunofluorescence microscopy 2.5–3.5 × 10^4^ cells/well were seeded in DMEMcplt into 24-well plates containing glass coverslips. At the indicated time points the cells were washed twice with PBS and fixed for 20 min by addition of 500 μL PBS containing 4% paraformaldehyde. After three washes with PBS, the cells were permeabilized with 0.2% Triton-X100 in PBS for 5 min. Permeabilized cells were blocked for one hour in PBS containing 0.01% Tween20 (PBS-T-0.01%) and 5% bovine serum albumin (BSA). The cells were incubated with the respective primary antibodies at appropriate concentrations for 2 h at room temperature. After three washes, the cells were incubated with the respective AlexaFluor (488, 568)-conjugated anti-mouse or anti-rabbit secondary antibodies (Life Technologies), respectively, diluted in PBS-T-0.01% containing 5% BSA. After three washes the nuclear DNA was stained with DAPI (Sigma-Aldrich) for 10 min. Finally, the coverslips were mounted on slides with FluoromountG (SouthernBiotech, Birmingham, AL, USA). The images were acquired with a Nikon Eclipse Ti microscope (Nikon, Tokyo, Japan) or a Leica SP8 (Leica, Wetzlar, Germany) confocal microscope. Western blotting was performed exactly as described earlier [27].

## 3. Results

### 3.1. In Silico Prediction of CEPs and Assembly of Synthetic Full Length ZIKV cDNAs

We focused on the development of infectious clones for two different ZIKV strains: MR766, which is a historical strain isolated from a rhesus monkey in 1947 [28] and H/PF/2013, a clinical isolate obtained in 2013 from a patient returning from French Polynesia [29]. Nucleotide sequences of the clones were based on the reference sequence DQ859059 for MR766 and KJ776791 for H/PF/2013. In addition, we determined the nucleotide sequences of these two virus strains that we propagated once in C6/36 mosquito cells and once in VeroE6 cells. We found that the H/PF/2013 isolate cultured in our cells was almost identical to the reference sequence with the exception of one nucleotide exchange resulting in an E1399Q amino acid substitution residing in NS2B, while the MR766 isolate differed by four nucleotide changes, two of them leading to E2197G and T3078A amino acid substitutions, residing in the NS4A and the NS5 coding region, respectively.

To assemble the complete genomes of these two strains, we introduced the mutations found in the viruses propagated in our laboratory and inserted in addition several silent nucleotide substitutions removing or creating restriction sites for convenient DNA cloning (Tables S1 and S2). These two sequences were dissected into 4 fragments that were generated by DNA synthesis (Figure 1A,B). The synthetic DNA fragments were assembled and inserted into a pFK-based vector via unique restriction sites [23]. A T7 promoter was inserted upstream of the ZIKV-5′UTR to allow for in vitro transcription of viral RNA. However, while fragments #2–#4 could be combined and amplified in *E. coli* with ease, insertion of fragment #1 repeatedly failed as we were not able to propagate a full-length ligation product. We reasoned that toxicity associated with fragment #1 might be the reason for our failure. In fact, for ZIKV it has been hypothesized that translation products generated from transcripts initiated at CEPs present in the structural region and NS1 might cause toxicity in bacteria [13]. Therefore, we decided to inactivate these bacterial promoters, a strategy successfully applied to the molecular cloning of DENV2 and JEV [11] and analysed our ZIKV sequences by using the promoter prediction tool from the Berkeley Drosophila Genome Project [20,21]. For MR766, 12 putative CEPs with a score >0.85 were detected within the first 2664 nucleotides (Figure 1A,B; Figure S1). By contrast, only eight putative CEPs were predicted within the first 2683 nucleotides of the H/PF/2013 genome (Figure 1B; Figures S1 and S2). To inactive these CEPs, nucleotide substitutions were inserted affecting the -10 region (Pribnow/ Schaller box) and/or the −35 region of all but one putative promoter. We did not alter the CEPs in the 5′ untranslated region (5′UTR) of our ZIKV strains because they contain complex RNA structures essential for RNA replication [22]. In total, 18 point mutations were introduced for MR766 and 12 for H/PF/2013 (Figures S1 and S2). For both strains, the modified sequence of fragment #1 was generated synthetically and inserted into the corresponding preassembled ZIKV constructs containing fragments #2–#4 without notable problems (Figure 1A,B). To confirm the stability of these synthetic full length ZIKV (synZIKV) cDNAs, the plasmids were passaged five times in *E. coli* and 5 clones of passage 5 were analysed by analytical restriction digest in comparison to the parental clone (Figure 1C). No obvious changes of the restriction patterns were found. Importantly, nucleotide sequences of the ZIKV genomes isolated after five bacterial passages were identical to the original genome and matched exactly the one generated in silico. Together, this result demonstrates that the synZIKV cDNA clones contained in the single bacterial plasmid vector are stable and that CEP silencing is a very simple and versatile approach to overcome stability problems of difficult-to-clone sequences.

### 3.2. Functionality of Full-Length synZIKV Wild-Type Genomes

**Figure 2:**
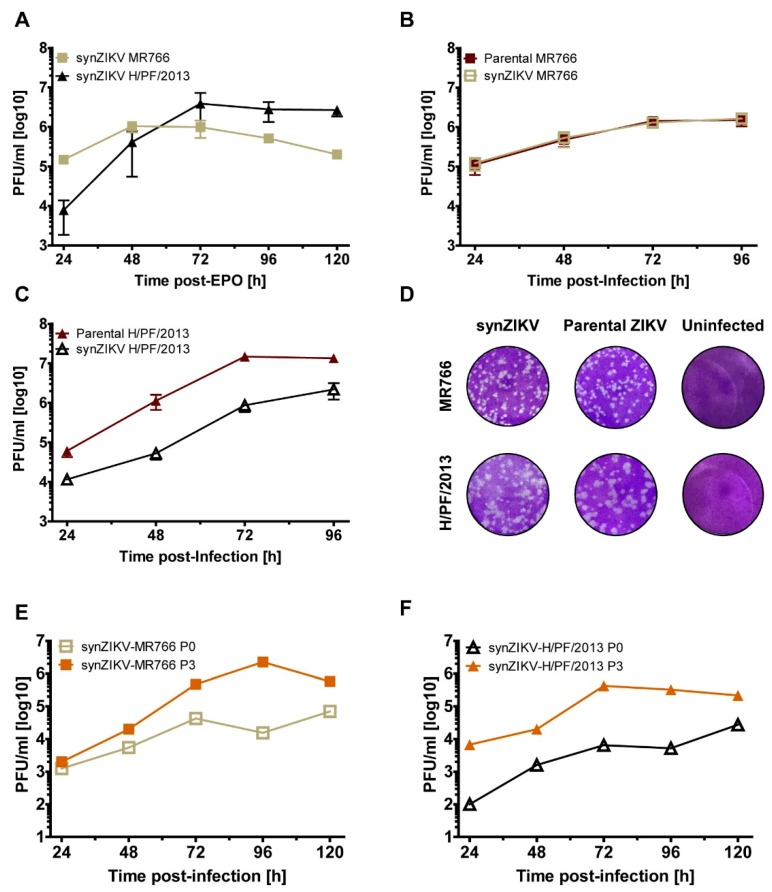
Replication kinetics of viruses obtained with the full-length synZIKV clones. (**A**) Replication kinetics of the two synZIKV clones as determined by plaque assay. VeroE6 cells were transfected with in vitro transcribed synZIKV RNAs and virus contained in culture supernatant at different time points after transfection was measured. Mean ± SEM of two independent experiments is shown. (**B**,**C**) Comparison of replication kinetics of synZIKV and parental viruses. Huh7 cells were infected with either ZIKV using a multiplicity of infection (MOI) of 1. Supernatants from infected cells were harvested at indicated times post-infection and titres were determined by plaque assay. Mean ± SEM of three independent experiments is shown. (**D**) Comparison of plaque morphology of synZIKV and the parental viruses. (**E**,**F**) Replication kinetics of passaged synZIKVs. Virus stocks were prepared as described in Materials and methods (P0). Huh7 cells were infected with MOI = 0.1 of P0 virus, cell culture supernatants were collected 72 h post-infection (P1) and passaged two more times by infection of Huh7 cells (P2–P3) in 72 h intervals. Huh7 cells were then infected using a MOI of 0.01 of P0 and P3 virus, respectively and virus titres were measured at indicated time points by plaque assay.

With the aim to determine functionality of the cloned full length synZIKV genomes VeroE6 cells were transfected with in vitro transcripts of synZIKV-MR766 and synZIKV-H/PF/2013. Peak virus titres of about 10^6^ plaque-forming units (PFU/mL) were detected in cell culture supernatants; maximum titres were reached faster by the MR766 strain than the HP/F/2013 strain arguing for different replication kinetics (Figure 2A). A comparison of the replication kinetics of the two synZIKV strains with the parental strains in Huh7 cells revealed virtually identical viral fitness in the case of the MR766 strain (Figure 2B). By contrast, wild-type synZIKV-H/PF/2013 replication was attenuated in this cell line relative to the parental H/PF/2013 strain but titres still reached ~10^6^ PFU/mL (Figure 2C). Similar results were obtained after infection with lower MOI [30]. Irrespective of that, plaque morphology was well comparable between the two synZIKVs and their parental strains (Figure 2D). The synMR766 strain formed smaller and more defined plaques, whereas synZIKV-H/PF/2013 formed large more diffuse and heterogeneous plaques but in both cases just like the corresponding WT strains (Figure 2D).

To determine whether passaging in cell culture could increase the fitness of our synZIKVs, we performed three serial passages in Huh7 cells and compared the replication kinetics of P0 and P3 viruses (Figure 2E,F). For both strains titres obtained with passaged viruses were higher than the ones of the corresponding P0 stock arguing for rapid adaptation of synZIKVs to cell culture conditions. Whether distinct adaptive mutations or the viral quasispecies in P3 virus cultures were responsible for increased fitness remains to be determined.

### 3.3. Replication and Stability of synZIKV Luciferase Reporter Virus Genomes

**Figure 3:**
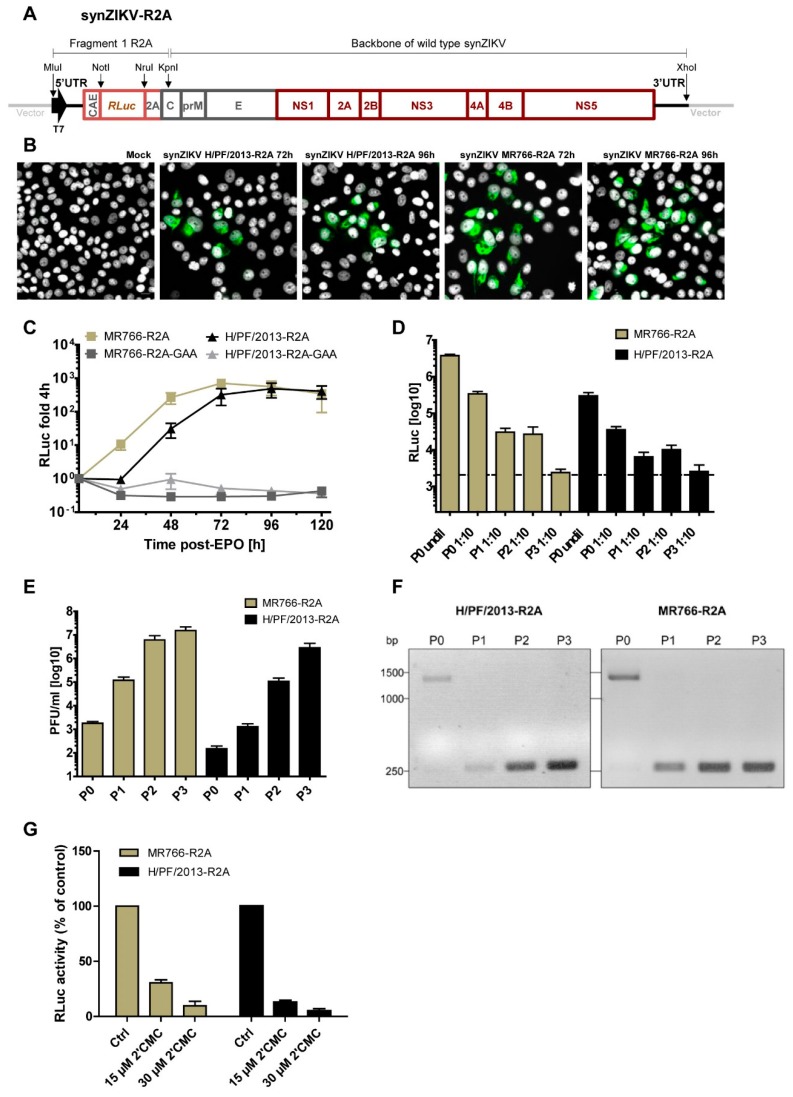
Construction and characterization of synZIKV-R2A reporter virus genomes. (**A**) Schematic representation of the synZIKV-R2A reporter virus genomes. For both strains the R2A reporter cassette (light red) was inserted into the wild-type pFK-synZIKV plasmids via MLuI/KpnI restriction sites. The NotI/NruI sites flanking the *RLuc* gene allow for the exchange of the reporter gene. (**B**) Immunofluorescence analysis of VeroE6 cells transfected with synZIKV-R2A in vitro transcripts. Cells were grown on coverslips, fixed 72 h and 96 h after transfection and stained with E-specific antibody (green). Nuclear DNA was counterstained with DAPI (grey). Scale bar = 15 μm. (**C**) Replication kinetics of the synZIKV-R2A reporter viruses in VeroE6 cells. After electroporation (EPO) cells were harvested at given time points and RLuc activity was determined. Values were normalized to the 4 h-value reflecting transfection efficiency. Mean ± SEM of three independent experiments is shown. Replication deficient mutants containing two mutations affecting the active site of the RNA-dependent-RNA polymerase in NS5 (GAA) served as negative controls. (**D**) VeroE6 cells were transfected with synZIKV-R2A RNAs, cell culture supernatants were collected 72 h post- transfection (P0) and passaged three times by infection of VeroE6 cells (P1-P3) in 72 h intervals. Culture supernatants obtained from each passage were used to inoculate Huh7 cells. In the case of supernatant obtained directly from transfected VeroE6 cells (P0), Huh7 cells were inoculated with undiluted (undil) or 1:10 diluted supernatant. After 72 h cells were harvested and RLuc activity in cell lysates was determined. Mean ± SEM from two independent experiments is shown. (**E**) Virus titres as determined by plaque assay for each synZIKV-R2A passage; values are mean ± SEM of two independent experiments. (**F**) Stability of the reporter gene. SynZIKV-R2A viruses released into culture supernatants were harvested after each passage as described in panel D, RNA was isolated and the region encompassing the RLuc coding sequence was amplified by using random hexamer primers for reverse transcription and specific primers for subsequent PCR. The ~1350 bp long DNA fragment in the P0 virus sample corresponds to the reporter gene, while the ~250 bp long fragment corresponds to the WT sequence. (**G**) Antiviral assay using synZIKV-R2A viruses. VeroE6 cells were inoculated with a 1:10 dilution of a P0 stock and one hour later the medium was replaced with DMEM containing the indicated amount of 2′CMC. RLuc activity was measured in cell lysates 72 h post-infection. Mean ± SEM from two independent experiments is shown.

Reverse genetic systems are powerful tools to study virus biology and pathogenesis but for some applications such as high-content screens reporter systems are superior because of the ease to measure virus replication in high-throughput formats [10]. We therefore manipulated both synZIKV genomes by insertion of a *Renilla luciferase* (*RLuc*) reporter gene (Figure 3A). In these genomes, the 5′ UTR is followed by the first 102 nts of the C-coding region containing an element that is required for genome circularization (CAE; capsid-circularization sequence). Downstream of the CAE we inserted the *RLuc* gene via engineered NotI and NruI restriction sites followed by the ribosome-skipping 2A sequence of the foot-mouth-disease virus (FMDV) to allow the release of the RLuc protein from the viral polyprotein. The functionality of these two synZIKV-R2A genomes was evaluated by electroporation of in vitro transcripts into VeroE6 cells. Virus replication was confirmed in transfected cells by E-specific immunofluorescence (Figure 3B) and quantified by measuring RLuc reporter activity in lysates of cells harvested at different time points after transfection (Figure 3C). As a reference, we constructed for both synZIKV-R2A clones a mutant, in which the catalytic site of the RNA-dependent-RNA-polymerase was inactivated by site-directed mutagenesis (mutants “GAA”). In addition, values were normalized to the 4 h-value reflecting transfection efficiency. For both synZIKV-R2A clones, robust replication was detected with faster kinetics in the case of the MR766 strain (Figure 3C) but comparable values detected at later time points after transfection (>96 h). However, as reported earlier [31], synZIKV-R2A viruses did not form plaques when harvested at early times post-transfection arguing that the insertion of the reporter gene caused attenuation [32].

Therefore, we determined the stability of the reporter virus genomes by multiple passaging of synZIKV-R2A particles collected 72 h post-transfection in VeroE6 cells. Virus contained in supernatants of each passage was used to infect Huh7 cells to determine RLuc reporter activity and plaque formation (Figure 3D,E). While RLuc activities were steadily decreasing with each passage and lost after passage 3 (Figure 3D), virus titres (PFU/mL) were increasing (Figure 3E) indicating a loss of the reporter gene and selection for synZIKV viruses with high replication fitness and plaque forming capability.

To support this assumption, synZIKV-R2A viruses released into culture supernatants were harvested after each passage, RNA was isolated and the region encompassing the RLuc coding sequence was amplified by RT-PCR (Figure 3F). In virus released from transfected cells (P0 supernatant), the amplicon had the expected size for the luciferase reporter gene (~1350 bp) although trace amounts of a smaller amplicon were also detected. However, already in P1 supernatant we could only amplify a fragment with a size expected for a WT clone (~250 bp). Sequence analysis of this PCR product confirmed a perfect match to the WT clone sequence, consistent with a rapid loss of the reporter gene. Nevertheless, by using virus contained in the supernatant of synZIKV-R2A transfected cells (i.e., P0 virus), virus replication was robustly detected and could be used for various assays, including antiviral drug testing. For instance, in line with a previous report [33], we found that the nucleoside 2′CMC strongly reduced the replication of both reporter viruses, thus demonstrating the versatility of our synZIKV system (Figure 3F).

### 3.4. SynZIKV Reporter Viruses Suitable for Live Cell Imaging

**Figure 4:**
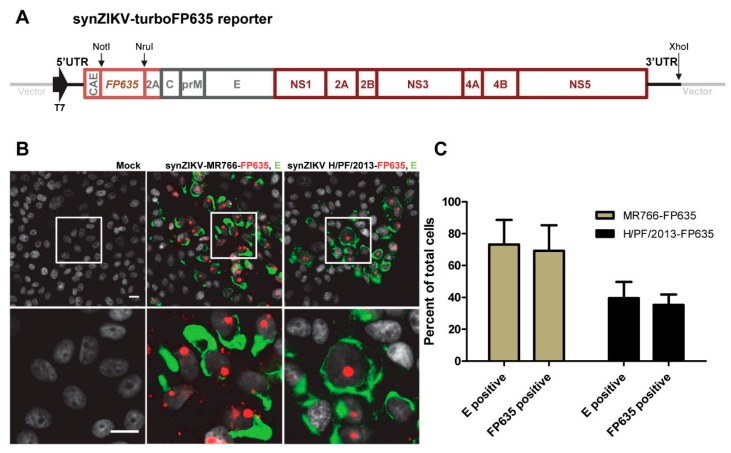
Construction and characterization of synZIKV-FP635 reporter viruses suitable for live cell imaging. (**A**) Schematic representation of the synZIKV-FP635 reporter genomes. The *FP635* gene fused at the 3′ end to the coding sequence of the SV40 NLS (not indicated) was inserted into the synZIKV constructs via NotI/NruI restriction sites. (**B**) Detection of E-antigen by immunofluorescence analysis of VeroE6 cells 96 h post-transfection with synZIKV-FP635 RNAs. The FP635 signal (red) was detected by its fluorescence. Note the accumulation of FP635 in distinct nuclear sites, most likely corresponding to nucleoli. Nuclear DNA was counterstained with DAPI (grey). Scale bar = 15 μm. (**C**) Quantification of E- and FP635-positive VeroE6 cells 96 h post-transfection of synZIKV-FP635 RNAs. Results show the mean from two independent experiments ± SEM. At least 150 cells per condition were counted.

In order to have at hand an easy to handle ZIKV system suitable for microscopy-based studies such as live-cell imaging, we replaced the *RLuc* reporter gene by a gene encoding the turbo far-red fluorescent protein FP635 (Figure 4A) [24]. Since ZIKV replicates in the cytoplasm we added a nuclear localization sequence (NLS) to the FP635 marker protein to avoid interference with imaging of cytoplasmic events. We used the well-studied NLS of the Simian Virus 40 (SV40) large T antigen that was fused to the C-terminus of FP635. Ninety-six hours after transfection with synZIKV-FP635 in vitro transcripts, cells were analysed by immune fluorescence to detect the E protein whereas FP635 was detected by its fluorescence (Figure 4B). Virtually all of the E-positive cells also expressed detectable amounts of FP635 (Figure 4B,C) suggesting that the synZIKV-FP635 reporter genomes are functional and allow the detection of infected cells just by means of the fluorescent marker protein. We noted that FP635 primarily accumulated within defined sub-nuclear regions, in line with a previous study reporting the accumulation of a GFP-SV40-NLS fusion protein in the nucleoli [34].

### 3.5. Sub-Genomic synZIKV Replicons

**Figure 5:**
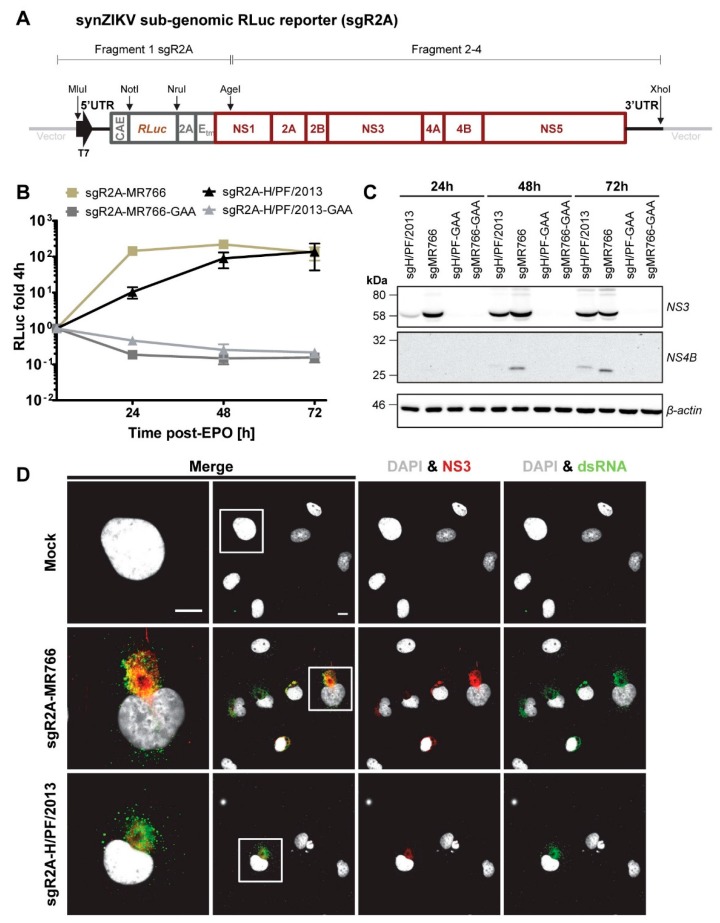
Properties of synZIKV sub-genomic reporter replicons. (**A**) Schematic representation of the synZIKV-sgR2A subgenomic reporter replicons. The reporter cassette (grey) was inserted into the synZIKV genomes via the MLuI and AgeI restriction sites and replaces the region encoding the structural proteins. (**B**) RLuc activity in Huh7 cells transfected with wild-type or replication-deficient (mutant GAA) synZIKV-sgR2A replicon RNAs measured at given times post-transfection. Shown RLuc values were normalized to the 4 h value to correct for transfection efficiency. Mean ± SEM of three independent experiments is presented. (**C**) Western blot showing the abundance of ZIKV NS3 and NS4B proteins in Huh7 cells transfected with synZIKV-sgR2A replicon RNAs. Cells were lysed at indicated times post-transfection and ZIKV-specific antibodies were used to detect viral proteins. β actin served as loading control. Numbers on the left refer to the positions of marker proteins that are given in kilodalton (kDa). (**D**) Immunofluorescence analysis of Huh7 cells 48 h post-transfection of synZIKV-sgR2A RNAs. Cells were stained with a dsRNA- (green) and a NS3-specific antibody (red). Nuclear DNA was stained with DAPI (grey). Scale bars = 15 μm. Boxed areas indicate regions that are shown in the left panels as enlargements.

In addition to full-length reporter viruses, sub-genomic replicons are powerful tools as they allow studying virus replication without biosafety concerns. Therefore, by using our full-length synZIKV molecular clones, we established sub-genomic RLuc reporter replicons. The overall construct design was analogous to the one of the synZIKV-R2A reporter genomes but the replicons lacked the region encoding the ZIKV structural proteins (Figure 5A). Replication of these sgR2A-synZIKV replicons was assessed in Huh7 cells (Figure 5B). RLuc activities detectable in the cell lysates at different time points after transfection correlated well with the amounts of ZIKV NS3 and NS4B proteins detectable by Western blot revealing that also in this case replication kinetics of the MR766 strain was faster than the H/PF/2013 strain (Figure 5C). NS3 as well as double-stranded RNA (dsRNA), a replication intermediate [22], were detectable by immunofluorescence (Figure 5C). As described for ZIKV-infected Huh7 cells, sgR2A-synZIKV-transfected cells had a kidney shaped nucleus [25]. This observation suggests that the non-structural proteins of ZIKV are sufficient to induce changes of nucleus morphology. In summary, these results show that sgR2A-synZIKV replicons are functional and induce morphological changes resembling a ZIKV infection.

## 4. Discussion

Here we describe a straightforward and very simple approach to establish a ZIKV reverse genetics system. The key feature is to remove CEPs, responsible for genome instability [9], by using in silico prediction with an open-access online tool and subsequent elimination in the virus sequence by silent nucleotide substitutions. This strategy was successfully applied to two different ZIKV strains—MR766 and H/PF/2013—for which construction of full-length cDNA clones has failed in the past [13,15,17,18] (A.P. and R.B., unpublished). Our approach allows the amplification of functional ZIKV infectious clones on a single, low-copy plasmid, which is superior to time consuming and error prone multi-vector systems reported earlier [15,16,17,18,19]. Moreover, in vitro transcripts generated from our synZIKV clones are infectious, thus mimicking an infection better than DNA-launched systems requiring nuclear transcription of the viral RNA genome. Finally, the possibility to stably propagate the ZIKV genome in a pBR-derived vector circumvents several disadvantages inherent to the use of bacterial artificial chromosome (BAC) systems, such as low DNA yield and complicated procedures to introduce mutations into the genome [35,36,37].

We did not observe a difference in replication dynamics between the wild-type synZIKV-MR766 molecular clone and the parental MR766 virus, suggesting that the molecular clone fully recapitulates the properties of the parental strain. Also, the plaque morphologies produced by both synZIKV-MR766 and synZIKV-H/PF/2013 closely resembled those of reference viruses. However, we observed that the synZIKV-H/PF/2013 molecular clone was attenuated (Figure 2E). The reasons underlying the reduced fitness are currently unknown. Although the sequence of the synZIKV-H/PF/2013 clone was modified to silence the CEPs and introduce unique restriction sites, these changes are unlikely to contribute to decreased viral fitness as all of the inserted mutations were silent and the regions known to contain regulatory RNA elements were omitted. We note however, that the attenuation observed by us is in line with the study of Widman and co-workers who constructed an H/PF/2013 molecular clone by using an in vitro ligation strategy and observed a similar degree of attenuation [15]. The fitness difference between the synZIKV-H/PF/2013 clone and the reference strain might be due to the genetic homogeneity of the molecular clone whereas the genome population of the H/PF/2013 isolate most likely is more heterogeneous, thus allowing for faster adaption to the cell culture conditions. Additionally, studies on poliovirus and influenza viruses showed that individual variants within viral quasispecies can cooperate to increase the fitness of the total virus population [38,39]. For MR766 this might not apply because this virus has been well adapted to cell culture conditions through intensive passaging since its isolation in 1947 [7]. This adaptation probably is already reflected in the cDNA sequence that served as reference for our clone (DQ859059). Nevertheless, fitness of both synZIKVs could be increased by cell culture passaging, arguing for rapid adaptation of both synZIKV strains and selection for variants with fitness even higher than the parental strains. Although we do not know whether distinct mutations in these synZIKV genomes or the -most likely- higher genetic heterogeneity in P3 stocks account for increased fitness, the use of extensively cell culture passaged virus is less desirable as it might have altered in vivo properties that are not necessarily detectable in vitro. Therefore, it is preferable to work with strains of low passage and defined sequence, which is the case with molecular clones as described here. Moreover, we note that the MR766 strain belongs to the early ZIKV isolates whereas H/PF/2013 is a more recent clinical strain that is closely related to strains isolated during the ZIKV epidemic in Brazil. For instance, the HP/F/2013 strain that we constructed has ~99.5% nucleotide sequence identity with the PE243 strain isolated from a Brazilian patient in 2015 (accession number KX197192) and only one amino acid change. Thus, our synZIKV clones should be useful for comparative studies between historical and contemporary ZIKV strains.

Owing to the reduction of replication fitness by the insertion of the *RLuc* reporter gene this ZIKV reporter virus was rather unstable and reporter-less variants with higher replication fitness were rapidly enriched during cell passage and already after one passage WT was the predominant species. This rapid deletion might be due to recombination occurring in *E. coli* during plasmid amplification and being facilitated by the partial duplication of the capsid coding region (i.e., the first 103 nts containing important *cis*-acting sequences of ZIKV), up- and downstream of the reporter gene. Alternatively, recombination might occur during virus propagation in cell culture. In any case, owing to higher fitness the WT virus rapidly out-competes the reporter virus and becomes the predominant species. Although this problem can be overcome by using ZIKV RLuc reporter viruses contained in culture supernatant of transfected (producer) cells, in which WT virus was not detected (Figure 3D,F), long-term propagation is not possible, which is a limitation when large stocks of reporter viruses are required. Therefore, further attempts are required to stabilize the inserted reporter gene, for example, by altering the sequences flanking the reporter gene, or inserting it into another region of the ZIKV genome. An alternative strategy might be the use of *trans*-complemented particles as we have developed for hepatitis C virus and DENV [40,41]. In this case the subgenomic replicon is transfected into a packaging cell line stably expressing the structural proteins. Virus-like particles are released from these cells that retain infectivity but contain the subgenomic RNA, thus requiring only low biosafety level. Importantly, since the size of the subgenomic replicon is much smaller than the complete genome it allows the insertion of rather long heterologous sequences without exceeding the size of the full-length genome.

In summary, this study reports a comprehensive toolbox for ZIKV research and an easy ZIKV cloning strategy that is based on CEP silencing, initially described for DENV2 and JEV [11]. As continuing globalization supports spreading of flaviviruses and their arthropod hosts, it is possible that outbreaks of other poorly characterized flaviviruses might occur in the future. Therefore, the strategy described here for ZIKV should allow the rapid construction and stable propagation of functional molecular clones of potentially emerging flaviviruses and other difficult-to-clone viruses.

## Supplementary Materials

The following are available online at http://www.mdpi.com/1999-4915/10/7/368/s1. Figure S1: Results of in silico prediction of cryptic prokaryotic promoters in the MR766 sequence. Figure S2: Results of in silico prediction of cryptic prokaryotic promoters in the H/PF/2013 sequence. Table S1: Modified restriction sites in the MR766 syn-sequence. Table S2: Modified restriction sites in the H/PF/2013 syn-sequence.
