# Supplementary material for: A Reverse Genetics System for Zika Virus Based on a Simple Molecular Cloning Strategy
Source: Viruses. 2018 Jul 12;10(7):368. doi: 10.3390/v10070368 (PMC6071187; doi:10.3390/v10070368)
Supplement: Supplementary file 1 [file viruses-10-00368-s001.pdf]

Supplementary Materials:

synZIKV H/PF/2013

#	Position		Sequences	Score
**1**	34-83	Original	ttcgagtttgaagcgaaagctagcaacagtatcaacaggtTttattttgg	0,98
		Modified	Not modified due to RNA secondary structures	
**2**	905-954	Original	gcttttgggaagctcaacgagccaaaaagtcatatacttgGtcatgatac	0,98
		Modified	gcttCtgggaagctcaacgagccaaaaagtcatCtacttgGtcatgatac	
**3**	1432-1481	Original	tcgttaatgacacaggacatgaaactgatgagaatagagcGaaggttgag	0,97
		Modified	tcgttaatgaTacaggacatgaaactgatgagaaCagagcGaaggttgag	
**4**	1836-1885	Original	ggccacttgaaatgtcgcctgaaaatggataaacttagatTgaagggcgt	0,96
		Modified	ggccacCtgaaatgtcgcctgaaaatggaCaaacttagatTgaagggcgt	
**5**	2104-2153	Original	tggaacttgatccaccatttggggactcttacattgtcatAggagtcggg	0,86
		Modified	tggaactCgatccaccatttggggactcttacattgtcatAggagtcggg	
**6**	2205-2254	Original	gcatttgaagccactgtgagaggtgccaagagaatggcagTcttgggaga	0,94
		Modified	gcattCgaagccactgtgagaggtgccaagagGatggcagTcttgggaga	
**7**	2341-2390	Original	cattgtttggaggaatgtcctggttctcacaaattctcatTggaacgttg	0,99
		Modified	cattgttCggaggaatgtcctggttctcacaGattctcatTggaacgttg	
**8**	2560-2609	Original	acgttgaagcctggagggaccggtacaagtaccatcctgaCtcccccgt	0,88
		Modified	acgtCgaagcctggagggaccggtacaagtaccatcctgaCtcccccgt	

X exchanged nucleotides for CEP silencing **X +1 nt of *in silico* predicted CEP (program output)**

Figure S1: Results of in silico prediction of cryptic prokaryotic promoters in the MR766 sequence.

synZIKV MR766

#	Position	Sequences		Score
1	34-83	Original	ttcgagtttgaagcgagagctaacaacagtatcaacaggtTtaatttgga	0,98
		Modified	Not modified due to RNA secondary structures	
2	353-402	Original	aaatttaagaaagatcttgctgccatgttgagaataatcaAtgctaggaa	0,97
		Modified	aaatttaagaaagatcttgctgccatgttgagGataatcaAtgctaggaa	
3	692-741	Original	acttgggttgtgtacggaacctgtcatcataaaaaaggtgAagcacggcg	0,94
		Modified	acGtgggttgtgtacggaacctgtcatcaCaaaaaaggtgAagcacggcg	
4	781-830	Original	gaaattgcaaacgcggtcgcagacttggctagaatcaagaGaatacacaa	0,97
		Modified	gaaattAcaaacgcggtcgcagacttggctGgaatcaagaGaatacacaa	
5	904-953	Original	gcttttgggaagctcgacgagccaaaaagtcatatacttgGtcatgatac	0,97
		Modified	gcttttAggaagctcgacgagccaaaaagtcatTtacttgGtcatgatac	
6	1215-1263	Original	cctaccttgacaagcaatcagacactcaatatgtttgcaaAagaacattg	0,96
		Modified	cctacctCgacaagcaatcagacactcaatatgtCtgcaaAagaacattg	
7	1657-1706	Original	tccacattggaacaacaaggaggcattagtggaattcaagGacgcccacg	0,94
		Modified	tccacaCtggaacaacaaggaggcattagtggaGttcaagGacgcccacg	
8	1780-1829	Original	ggctgagatggatggtgcaaagggaaggctattctctggcCacttgaaat	0,88
		Modified	ggctgagatggatggtgcaaagggaaggctGttctctggcCacttgaaat	
9	2048-2097	Original	gtgattactgaaagcactgagaattcaaagatgatgttggAgctcgaccc	0,94
		Modified	gtgatCactgaaagcactgagaattcaaagatgatgttggAgctcgaccc	
10	2111-2160	Original	tcttacattgtcataggagttggggataagaaaatcacccAtcactggca	0,94
		Modified	tcttacatCgtcataggagttggggataagaaGatcacccAtcactggca	
11	2186-2235	Original	gcatttgaagccactgtgagaggcgctaagagaatggcagTcctgggggа	0,95
		Modified	gcattCgaagccactgtgagaggcgctaagagGatggcagTcctgggggа	
12	2541-2590	Original	acgttgaagcctggagggaccggtacaagtaccatcctgaCtcccctcgt	0,88
		Modified	acgtCgaagcctggagggaccggtacaagtaccatcctgaCtcccctcgt	

X exchanged nucleotides for CEP silencing **X +1 nt of *in silico* predicted CEP (program output)**

Figure S2: Results of in silico prediction of cryptic prokaryotic promoters in the H/PF/2013 sequence.

Table S1. Modified restriction sites in MR766 syn-sequence.

Position of restriction site	Original codon within site	Changed codon within site	Enzyme	Added/ deleted	Comment
896	GCC	GCG	NruI	+	Methylated in dcm+/ dam+ bacteria
1748	GGA	GGC	NarI	+	None
3317	GGT	GGA	KpnI	-	One site remains
3845	CTG	CTA	NheI	-	One site remains
6387	AGG	CGG	XmaI	+	None
7769	GCT	GCG	AscI	+	None
8264	GGA	GGC	SpeI	-	One site remains

Table S2. Modified restriction sites in H/PF/2013 syn-sequence.

Position of restriction site	Original codon within site	Changed codon within site	Enzyme	Added/ deleted	Comment
711	GGA	GGT	KpnI	+	None
747	TCT	TCC	XbaI	-	One site remains
2581	AGG	CGG	AgeI	+	None
2712	GAG	GAA	SacI	-	One site remains
3858	AGC	AGT	SphI	-	One site remains
4323	GCC	GCA	SacII	-	One site remains
5752	AGC	AGT	SacI	-	One site remains
7737	GAG	GAA	EcoRI	+	None
8520	AGG	AGA	BamHI	-	One site remains
9120	TTT	TTC	XbaI	-	One site remains
